# Optimization of Ultrasound-Assisted Extraction of Total Flavonoids from *Dendranthema indicum* var*. aromaticum* by Response Surface Methodology

**DOI:** 10.1155/2019/1648782

**Published:** 2019-07-17

**Authors:** Lijie Zhong, Yi Liu, Bin Xiong, Lin Chen, Yaohua Zhang, Chao Li

**Affiliations:** ^1^Technology Center of China Tobacco Hubei Industrial LLC, Wuhan 430040, China; ^2^Key Laboratory of Analytical Chemistry for Biology and Medicine (MOE), College of Chemistry and Molecular Sciences, Wuhan University, Wuhan 430072, China; ^3^Key Laboratory of Coal Conversion and Carbon Materials of Hubei Province, College of Chemistry and Chemical Engineering, Wuhan University of Science and Technology, Wuhan 430081, China; ^4^College of Chemistry and Material Sciences, Guangxi Teachers Education University, Nanning 530001, China

## Abstract

*Dendranthema indicum* var. *aromaticum* is a new species with strong fragrance and is used as a herbal medicine by Chinese folks. The abundant flavonoids play important roles in its pharmacological activities. In this study, an ultrasound-assisted method was used to extract total flavonoids (TF) from *D. indicum* var. *aromaticum* by response surface methodology. A quadratic model was developed to optimize the extraction conditions, whose accuracy was verified by statistic analysis. Ethanol and acetic acid at the volume ratio of 70% : 2% were selected as the extract solvent. The optimized extraction conditions were as follows: extraction time, 40 min; solid/liquid ratio, 1 : 23 g/mL; and temperature, 60°C. This is the first report of an efficient and easy-operating method for TF extraction from *D. indicum* var. *aromaticum*. Besides, this study provides reference for future pharmacological research on *D. indicum* var. *aromaticum* and extraction of bioactive components from other herbs.

## 1. Introduction

*Dendranthema indicum* var*. aromaticum*, a new species with intense fragrance throughout the whole plant [1], inhabits in Shenongjia primitive forest of Hubei province in China. The essential oil extracted from *D. indicum* var*. aromaticum* has been reported to have antimicrobial and antioxidant activities [2] and is widely used as essence in tobacco, perfume, and cosmetics industries due to its natural aroma and bioactivities [3].

Besides its economic values, *D. indicum* var. *aromaticum* also has great medicinal values. Chinese folks use the plant to prevent cold and deal with headache, constipation, enteritis, coronary disease, and hypertension. It has been reported that *D. indicum* var*. aromaticum* contains abundant flavonoids [4]. According to epidemiological studies, dietary intake of flavonoids has a negative correlation with coronary heart disease risk in a dose-dependent manner [5, 6] and people with higher intake of total flavonoids (TF) are less likely to develop hypertension [7, 8]. Some flavonoids have been isolated from *D. indicum* var*. aromaticum*, such as luteolin, apigenin, and acacetin [4]. Among them, apigenin has been found to have anti-inflammatory, antidiarrhoea, and vasorelaxant pharmacological activities [9–11]. Acacetin can induce cell cycle arrest and apoptosis in a variety of human cancer cell lines [12, 13]. Although many studies have revealed the high pharmacological values of flavonoids in *D. indicum* var*. aromaticum*, there has been little research on the optimization of methods and conditions for the extraction of flavonoids from *D. indicum* var*. aromaticum*.

Various new methods have been developed and applied in the extraction of flavonoids in recent decades, such as enzyme-assisted extraction, membrane adsorption, and supercritical fluid extraction [14–16]. These newly developed methods have drawn much attention due to their prominent advantages such as high efficiency and purity of the product. However, the rigid extraction conditions, complex operations, high requirements of techniques and equipment, and high cost largely hinder the wide application of these methods in real production.

Ultrasound-assisted extraction is a popular method used in the extraction of bioactive compounds from plant materials. Wang et al. compared the microstructures of plant materials extracted with and without ultrasound treatment and found that ultrasound-assisted extraction helped to eliminate the outer pectin materials and expose the cell wall clearly under a scanning electron microscope after ultrasound treatment, suggesting that ultrasound-assisted extraction could achieve the same effect as enzyme-assisted extraction [17]. Ultrasound-assisted extraction not only enhances the fragmentation but also assists the release, diffusion, and dissolution of the components inside cells [18]. This method was developed on the basis of traditional extraction and can greatly shorten the extraction time. In addition, it does not require complex equipment or techniques, which largely decreases the production cost compared with other methods. Therefore, ultrasound-assisted extraction was employed in this study to extract flavonoids from *D. Indicum* var. *aromaticum*.

Central composite circumscribed (CCC) design is one of the design methods for response surface methodology which was originally described by Box and Wilson [19]. It can optimize the factors for desirable responses by building empirical models and is less laborious and time-consuming and more accurate than full-factorial design [20]. This study aims to seek the optimum conditions for the extraction of TF from *D. indicum* var. *aromaticum* by response surface methodology.

## 2. Materials and Methods

### 2.1. Plant Materials

*D. indicum* var. *aromaticum* plant materials were collected from Shennongjia area in Hubei province, China. The plant species was authenticated by Herbarium, Kunming Institute of Botany, Chinese Academy of Sciences, and a voucher specimen was deposited (No. Dendranthema 001). The overground part was dried and stored at 80°C.

### 2.2. Chemicals and Reagents

Ethanol (AR) and acetic acid (AR) were purchased from Sinopharm Chemical Reagent Co., Ltd (Shanghai, China). Aluminum chloride (AR, ≥99.0%) and potassium acetate (AR, ≥99.0%) were obtained from General-Reagent (Shanghai, China). Quercetin (HPLC, ≥95%) was purchased from Sigma-Aldrich (MO, USA).

### 2.3. Ultrasound-Assisted Extraction of TF

A certain volume of solvent was added to 0.5 g of grinded *D. indicum* var. *aromaticum* (Shanghai Lijian Machinery Co., Ltd, Shanghai, China), and then, the tubes were put into a 360 W ultrasound bath for TF extraction (KQ-600DB, 40 kHz, Kunshan Ultrasonic Instruments Co., Ltd, China). The temperature of the ultrasound bath was maintained by circulated water from external water bath. Samples were then centrifuged at 4°C and 10,000 *g* for 10 min (Allegra X-30R centrifuge, Bechman Coulter, Inc., California, USA). The supernatant was used for subsequent determination of TF content. All extraction experiments were conducted in triplicate.

### 2.4. Effect of Solvent on TF Extraction

Mixed solvents of ethanol (30%, 50%, and 70%) and acetic acid (2%, 5%, and 10%) were prepared in an orthogonal design. Different solvents (10 mL) were used to extract 0.5 g of plant materials at 40°C ultrasound bath for 30 min. The supernatant was collected after centrifugation as mentioned above.

### 2.5. Effect of Extraction Time on TF Extraction

Ethanol-acetic acid (70% : 2%, v/v) solvent (10 mL) was used to extract 0.5 g of plant materials at 40°C ultrasound bath for different time periods (20, 30, 40, 50, and 60 min). The supernatant was collected after centrifugation as mentioned above.

### 2.6. Effect of Solid/Liquid Ratio on TF Extraction

Ethanol-acetic acid (70% : 2%, v/v) solvent with different solid/liquid ratios (g/mL) (1 : 10, 1 : 15, 1 : 20, 1 : 25, and 1 : 30) was used to extract 0.5 g of plant materials at 40°C ultrasound bath for 30 min. The supernatant was collected after centrifugation as mentioned above.

### 2.7. Effect of Extraction Temperature on TF Extraction

Ethanol-acetic acid (70% : 2%, v/v) solvent (10 mL) was used to extract 0.5 g of plant materials at different temperatures (30, 40, 50, and 60°C) of ultrasound bath for 30 min. The supernatant was collected after centrifugation as mentioned above.

### 2.8. Conventional Extraction of TF

Plant samples were extracted with ethanol -acetic acid (70% : 2%, v/v) solvent by shaking (RH-Q shaker, Jintan Electronic, Inc., Jiangsu, China) under general conditions (solid/liquid ratio, 1 : 20 g/mL; temperature, 40°C; extraction time, 30 min or 24 h) and under optimized conditions (solid/liquid ratio, 1 : 23 g/mL; temperature, 60°C; extraction time, 40 min or 24 h). The supernatant was collected after centrifugation as mentioned above.

### 2.9. Determination of TF Extraction Rate

The TF extraction rate was measured by the aluminum chloride method as described in a previous study with modifications [21]. Briefly, 200 *μ*L of appropriately diluted extract, 600 *μ*L of 95% ethanol, 40 *μ*L of 10% (m/v) aluminum chloride, 40 *μ*L of 1 M potassium acetate, and 1.12 mL of ultrapure water were mixed. The mixture was then incubated at room temperature for 30 min after vortexing. The absorbance of the mixture was measured at 415 nm with a blank (200 *μ*L of extraction solvent instead of the extract) using a nucleic acid/protein analyzer (Beckman Coulter, DU 730, CA, USA). Quercetin was used to construct the calibration curve, and the results were expressed as quercetin equivalent (g QE/100 g DW).

### 2.10. Experimental Design and Statistical Analysis

The conditions for the extraction of TF from *D. indicum* var. *aromaticum* were adopted according to a central composite circumscribed (CCC) design. A three-variable and five-level CCC model was applied. The three independent variables were extraction time (min, *X*_1_), solid/liquid ratio (g/mL, *X*_2_), and extraction temperature (°C, *X*_3_). The independent variables and their levels for CCC design are shown in Table 1. A total of 20 experiments corresponding to eight factorial points, six axial points, and six central points were carried out in a random order (Table 2). Regression analysis was performed to fit the following quadratic polynomial model:

(1) $$\begin{array}{c} Y=\;\beta_{0}+\overset{3}{\underset{i=1}{\sum }}\beta_{i}X_{i}+\overset{3}{\underset{i=1}{\sum }}\beta_{ii}X_{i}^{2}+\overset{3}{\underset{i\neq j=1}{\sum }}\beta_{ij}X_{i}X_{j}, \end{array}$$

where *β*_*0*_ is a constant intercept, and *β*_*i*_, *β*_*ii*_, and *β*_*ij*_ are linear, quadratic, and interactive regression coefficients of the model, respectively. Statistical results and response surface plots were generated using Design Expert 10 software (Stat Ease Inc., Minneapolis, USA). *P* < 0.05 was considered to be statistically significant.

## 3. Results and Discussion

### 3.1. Selection of Extraction Solvent

The solubility of flavonoids could be affected by the polarity and property of the solvent used. Solvents commonly used for the extraction of phenolic compounds (including flavonoids) from botanical materials mainly include aqueous methanol, ethanol, and acetone [22, 23]. Previous studies have reported that the addition of small amounts of ethanol could quench the radical production and decrease the temperature inside the cavitation bubbles generated by the high frequency of ultrasound [24, 25]. Based on these reports and considering that ethanol is environmental friendly and relatively less toxic for human health, ethanol was chosen as the main organic solvent for TF extraction in this study. Acids, such as hydrochloric acid, formic acid, or acetic acid, are usually added to achieve a higher extraction efficiency according to previous studies [22, 23]. It is known that lower acidity results in lower toxicity, whether it is inorganic or organic acids. As a weak acid, acetic acid is not toxic at low concentrations, and it has been found that 5% acetic acid in water could increase the efficiency of phenolic antioxidant extraction [26]. Thus, we employed low concentrations of acetic acid combined with ethanol in an attempt to enhance the efficiency of flavonoid extraction in this study.

**Figure 1:**
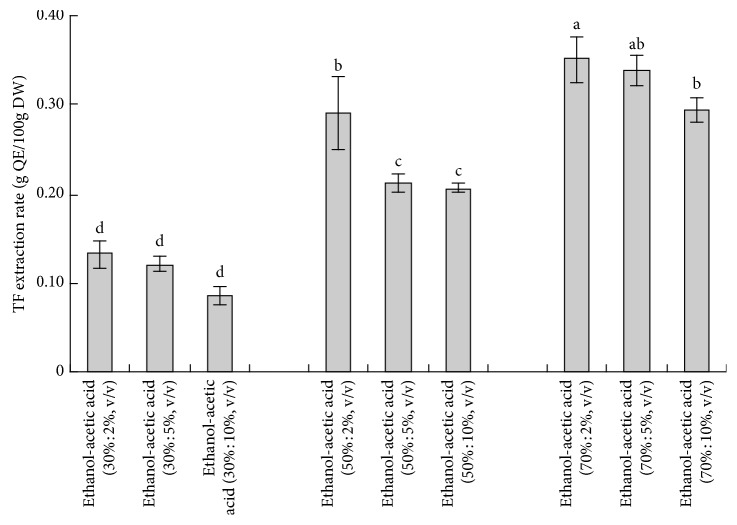
Effect of different combinations of ethanol and acetic acid in proportion on total flavonoids (TF) extraction rate.

Figure 1 shows the effects of different combinations of ethanol and acetic acid in proportion on TF extraction. In general, the TF amount extracted from *D. indicum* var*. aromaticum* was increased with increasing ethanol proportion (from 30% to 70%), and the addition of 2% acetic acid contributed to a higher TF extraction rate than the addition of 5% or 10% actetic acid. The extraction rate of ethanol and acetic acid at the ratio of 70% : 2% (v/v) was slightly higher than that at the ratio of 70% : 5% (v/v) and was significantly higher than that of other tested solvents (Figure 1). Therefore, ethanol and acetic acid ratio of 70% : 2% (v/v) was selected for further study.

### 3.2. Selection of the Ranges of Independent Variables

**Figure 2:**
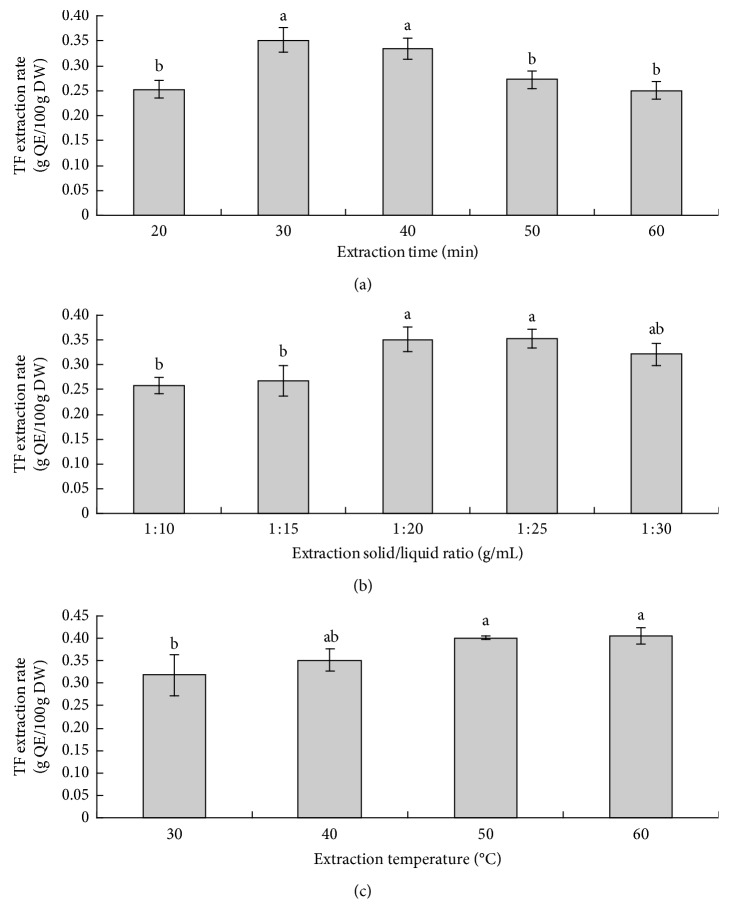
Effect of individual independent variables: (a) extraction time, (b) extraction solid/liquid ratio, and (c) extraction temperature on total flavonoids (TF) extraction rate.

Figure 2 shows the effects of individual independent variables of extraction time, extraction solid/liquid ratio, and extraction temperature on TF extraction. As shown in Figure 2(a), the TF extraction rate reached significantly higher levels at 30 and 40 min than at 20, 50, and 60 min, indicating that adequate ultrasound time could increase the yield of TF, but there is a risk of flavonoid degradation at excessive ultrasound extraction time [27, 28]. The TF extraction rate increased with the solid/liquid ratio increasing from 1 : 10 to 1 : 20 g/mL and then stayed stable at the ratio of 1 : 25 g/mL, followed by a slight decrease at the ratio of 1 : 30 g/mL (Figure 2(b)). As for the extraction temperature, the TF extraction rate increased from 30°C to 50°C and remained at a consistently high level until 60°C (Figure 2(c)). Chaaban et al. studied the thermal stability of flavonoids and found that flavonoids are more or less sensitive to heat treatment depending on their structures and aglycon flavonoids are easier to be degraded than glycosylated flavonoids under heat treatment [29]. Therefore, both excessive ultrasound time and high temperature should be avoided in flavonoid extraction. According to the effects of each independent variable on the TF extraction rate, the central point of the response surface model was set with the following conditions: extraction time, 30 min; solid/liquid ratio, 1 : 20 g/mL; and extraction temperature, 50°C. The subsequent response surface model covered the appropriate time range (20, 30, and 40 min), solid/liquid ratio range (1 : 10, 1 : 20, and 1 : 30 g/mL), and temperature range (40, 50, and 60°C).

### 3.3. Fitting of the Response Surface Model

A total of 20 experimental runs for optimizing the extraction conditions were designed and operated. Extraction conditions and their corresponding TF extraction rates are listed in Table 2. Multiple regression analysis was operated to fit the linear, interactive (2FI), quadratic, and cubic models. According to results of sequential model sum of squares (Table 3), the cubic model was found to be biased and both linear and quadratic models had lower *p* values (<0.0001). Model summary statistics in Table 4 show that the *R*^2^, adjusted *R*^2^, and predicted *R*^2^ values of the quadratic model (0.9814, 0.9647, and 0.9023, respectively) are higher than those of the linear model (0.8213, 0.7877, and 0.7295, respectively). The *R*^2^ values closer to 1 indicate better correlations between the variables. Therefore, the quadratic model was selected and used for further analysis.

To better predict the effects of different conditions on the extraction efficiency, a second-order polynomial mathematical equation with interaction terms was developed. The final equation obtained in terms of coded factors is shown as follows:

(2) $$\begin{array}{c} Y=0.39+0.037X_{1}+9.898\times 10^{-3}X_{2}+0.046X_{3}+2.814\times 10^{-4}X_{1}X_{2}-6.185\times 10^{-3}X_{1}X_{3}+3.692\times 10^{-3}X_{2}X_{3}-3.192\times 10^{-3}X_{1}^{2}-0.023X_{2}^{2}-0.013X_{3}^{2}. \end{array}$$

Analysis of variance (ANOVA) was operated to check the accuracy and fitness of the quadratic model, and the results are shown in Table 5. First of all, the high *F*-value (58.69) and the low *p* value (<0.0001) of the model indicate that the model can significantly represent the relationship between the response and independent variables [30, 31]. Secondly, the lack of fit of the developed quadratic model is insignificant relative to the pure error, and its *p* value of 0.3035 means a 30.35% chance of the occurrence of the lack of fit of *F*-value due to noise, indicating a good acceptability of the quadratic model. The estimated coefficients of each term in the model are also shown in Table 5. The linear term coefficients *X*_1_, *X*_2_, and *X*_3_, and quadratic term coefficients *X*_2_^2^ and *X*_3_^2^ are significant, suggesting that all three extraction parameters, including extraction time, solid/liquid ratio, and extraction temperature, significantly affect the productivity of flavonoids.

### 3.4. Diagnosis of the Model

**Figure 3:**
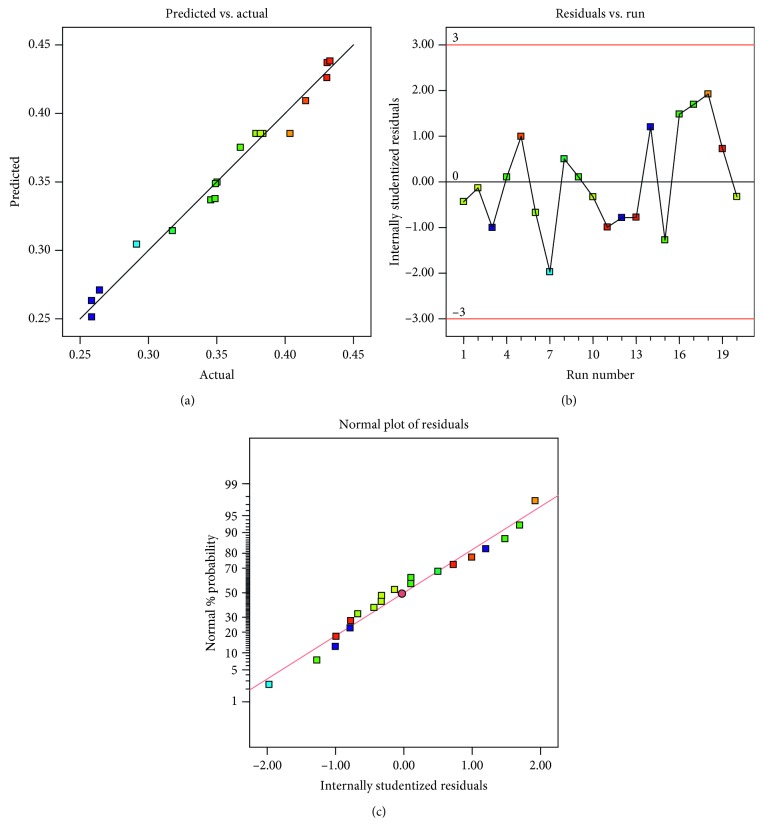
Diagnostic plots of the quadratic model. (a) Diagnosis of experimental and predicted values of TF extraction rate. (b) Plot of internally studentized residuals for TF extraction rate. (c) Normal probability plot for TF extraction rate.

In general, it is essential to check the accuracy of the model, because the fitting of response surface model may produce poor or misleading results [32]. The plot in Figure 3(a) was used to check the consistency between the predicted values and actual values. In theory, if the experimental results are exactly predicted by the model, the data points would be exhibited on a straight line. Hence, a data point closer to the straight line indicates that the predicted value is more approximate to the actual value. As shown in Figure 3(a), the data points were closely distributed around a straight line, indicating that a good correlation between the predicted and actual values was obtained from the response surface model.

In addition, the accuracy of the model could also be checked by internally studentized residuals (Figure 3(b)) and their normal probability (Figure 3(c)). In Figure 3(b), the maximum absolute value of the internal studentized residuals is around 2. Empirically, the model is acceptable if the absolute values of internally studentized residuals are lower than 3; otherwise, the model needs to be reconsidered. In Figure 3(c), the internally studentized residuals are distributed closely around a straight line and the values are normally distributed. By diagnosing the internally studentized residuals, the quadratic model fitting was confirmed to be satisfactory.

### 3.5. Optimization and Verification of Extraction Conditions

**Figure 4:**
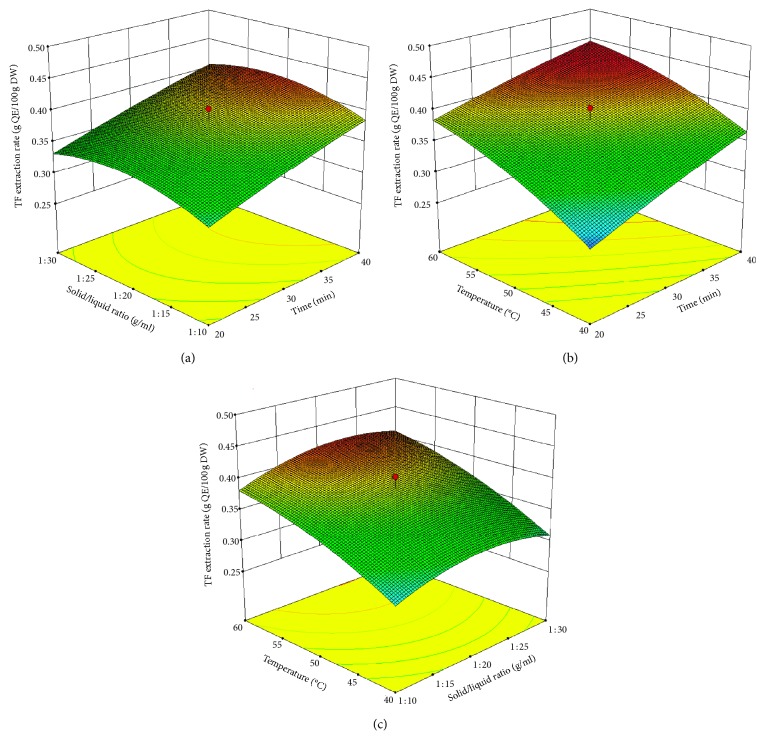
Response surface plots for TF extraction rate affected by (a) solid/liquid ratio and extraction time (extraction temperature was set at 50°C), (b) extraction temperature and time (solid/liquid ratio was set at 1 : 20), and (c) extraction temperature and solid/liquid ratio (extraction time was set at 20 min).

The effects of extraction time, solid/liquid ratio, and extraction temperature on TF extraction rate in *D. indicum* var. *aromaticum* were represented by response surface plots in Figure 4. In the three-dimensional response surface plots, the TF extraction rate was obtained with two continuous variables, and the other variable was set at zero level.

In Figure 4(a), when the temperature was set at zero level (50°C), the TF extraction rate increased with the extension of extraction time at a fixed solid/liquid ratio, particularly at the ratios ranging from 1 : 20 to 1 : 25 g/mL. The effects of extraction time and temperature on the TF extraction rate are shown in Figure 4(b). When the solid/liquid ratio was set at zero level (1 : 20), the TF extraction rate increased with increasing extraction time and extraction temperature. A combination of longer extraction time (20–40 min) and higher temperature (40–60°C) contributed to a higher TF extraction rate.  Figure 4(c) shows the effects of solid/liquid ratio and extraction temperature on the TF extraction rate when the extraction time was set at zero level (30 min). A relatively higher TF extraction rate was found under the combination of higher temperature (55–60°C) and appropriate solid/liquid ratio (1 : 20–1 : 25 g/mL).

The purpose of this study was to optimize an extraction method for high productivity of TF from *D. indicum* var. *aromaticum*. The optimum extraction conditions were obtained from the developed response surface model: extraction time, 40 min; solid/liquid ratio, 1 : 23 g/mL; and extraction temperature, 60°C (Table 6). Rechecking experiment was operated under the optimum conditions to confirm the accuracy of the predicted results (0.448 g QE/100g DW). A mean value of 0.424 ± 0.029 g QE/100 g DW was obtained from real experiments. The good correlation between the predicted values and experimental results confirmed that the response surface model developed in this study is reliable to represent the actual effects of the optimized extraction conditions.

### 3.6. Comparison of the Extraction Effects with and without Ultrasound Treatment

The TF extraction efficiencies with ultrasound treatment were compared with those obtained without ultrasound treatment (conventional extraction), and the results are shown in Table 7. Whether under general conditions or optimized conditions, the TF extraction rates with ultrasound treatment were about 10-folds higher than those obtained without ultrasound treatment. In addition, ultrasound-assisted extraction for 30 min resulted in an even higher TF yield than conventional extraction for 24 h. These results clearly demonstrate that ultrasound-assisted extraction can enhance the extraction efficiency and greatly reduce the extraction time.

## 4. Conclusion

In the present study, response surface methodology was successfully applied to optimize the extraction conditions of TF from *D. indicum* var. *aromaticum*. Regression analysis, ANOVA results, and model diagnosis results all verified the accuracy of the model. Optimum conditions were obtained from the developed quadratic equation, and the experimental results under optimum conditions were pretty close to the predicted values. The extraction efficiency of ultrasound-assisted extraction was proved to be greatly higher than that of conventional extraction. This study provides technical reference for future pharmacological research on the newly found species *D. indicum* var. *aromaticum*, as well as for the application of response surface methodology in the extraction of bioactive components from other herbs.

## Figures and Tables

**Table 1:** Independent variables and their levels in central composite circumscribed (CCC) design.

Symbol	Independent variable	Factor level				
		−1.68	−1	0	1	1.68
*X*_1_	Extraction time (min)	13	20	30	40	47
*X*_2_	Solid/liquid ratio (g/mL)	1 : 03	1 : 10	1 : 20	1 : 30	1 : 37
*X*_3_	Extraction temperature (°C)	33	40	50	60	67

**Table 2:** Five-level and three-variable central composite circumscribed (CCC) design for total flavonoids (TF) extraction in *D. indicum* var. *aromaticum* and observed responses.

Run	*X*_1_ (min)	*X*_2_ (g/mL)	*X*_3_ (°C)	TF extraction rate (g QE^b^/100 g DW)
1	20 (−1)	1 : 10 (−1)	40 (−1)	0.2586
2	40 (1)	1 : 10 (−1)	40 (−1)	0.3457
3	20 (−1)	1 : 30 (1)	40 (−1)	0.2385
4	40 (1)	1 : 30 (1)	40 (−1)	0.3104
5	20 (−1)	1 : 10 (−1)	60 (1)	0.4192
6	40 (1)	1 : 10 (−1)	60 (1)	0.4452
7	20 (−1)	1 : 30 (1)	60 (1)	0.3675
8	40 (1)	1 : 30 (1)	60 (1)	0.4109
9	13 (−1.68)	1 : 20 (0)	50 (0)	0.3177
10	47 (1.68)	1 : 20 (0)	50 (0)	0.4330
11	30 (0)	1 : 03 (−1.68)	50 (0)	0.2115
12	30 (0)	1 : 37 (1.68)	50 (0)	0.4189
13	30 (0)	1 : 20 (0)	33 (−1.68)	0.2644
14	30 (0)	1 : 20 (0)	67 (1.68)	0.4307
15	30 (0)	1 : 20 (0)	50 (0)	0.3811
16	30 (0)	1 : 20 (0)	50 (0)	0.3822
17	30 (0)	1 : 20 (0)	50 (0)	0.3840
18	30 (0)	1 : 20 (0)	50 (0)	0.4037
19	30 (0)	1 : 20 (0)	50 (0)	0.3788
20	30 (0)	1 : 20 (0)	50 (0)	0.3321

*Note*. Numbers in brackets are coded independent variables. ^b^Quercetin equivalent.

**Table 3:** Sequential model sum of squares.

Source	Sum of squares	Degree of freedom	Mean square	*F*-value	Prob. > *F*	
Mean	2.57	1	2.57			
Linear	0.05	3	1.63*E* − 02	24.51	<0.0001	
2FI	4.16*E* − 04	3	1.39*E* − 04	0.18	0.91	
Quadratic	9.11*E* − 03	3	3.04*E* − 03	27.48	<0.0001	Suggested
Cubic	6.76*E* − 04	4	1.69*E* − 04	2.36	0.17	Biased
Residual	4.29*E* − 04	6	7.15*E* − 05			
Total	2.63	20	0.13			

**Table 4:** Model summary statistics.

Source	Standard deviation	*R* ^2^	Adjusted *R*^2^	Predicted *R*^2^	PRESS	
Linear	2.58*E* − 02	0.8213	0.7877	0.7295	0.0161	
2FI	2.80*E* − 02	0.8283	0.7490	0.6024	0.0236	
Quadratic	1.05*E* − 02	0.9814	0.9647	0.9023	0.0058	Suggested
Cubic	8.46*E* − 03	0.9928	0.9772	0.9595	0.0024	Biased

**Table 5:** Analysis of variance (ANOVA) for the response surface quadratic model.

Source	Estimated coefficients	Sum of squares	Degree of freedom	Mean square	*F*-value	Prob. > *F*
Model	0.3853	5.83*E* − 02	9	6.48*E* − 03	58.69	<0.0001
*X*_1_	3.68*E* − 02	1.85*E* − 02	1	1.85*E* − 02	167.40	<0.0001
*X*_2_	9.90*E* − 03	1.34*E* − 03	1	1.34*E* − 03	12.11	0.0059
*X*_3_	4.61*E* − 02	2.90*E* − 02	1	2.90*E* − 02	262.52	<0.0001
*X*_1_ *X*_2_	2.81*E* − 04	6.33*E* − 07	1	6.33*E* − 07	0.01	0.9411
*X*_1_ *X*_3_	−6.19*E* − 03	3.06*E* − 04	1	3.06*E* − 04	2.77	0.1270
*X*_2_ *X*_3_	3.69*E* − 03	1.09*E* − 04	1	1.09*E* − 04	0.99	0.3439
*X*_1_^2^	−3.19*E* − 03	1.47*E* − 04	1	1.47*E* − 04	1.33	0.2758
*X*_2_^2^	−2.27*E* − 02	7.42*E* − 03	1	7.42*E* − 03	67.16	<0.0001
*X*_3_^2^	−1.30*E* − 02	2.44*E* − 03	1	2.44*E* − 03	22.12	0.0008
Residual		1.10*E* − 03	10	1.10*E* − 04		
Lack of fit		6.84*E* − 04	5	1.37*E* − 04	1.63	0.30
Pure error		4.21*E* − 04	5	8.42*E* − 05		
Cor. total		5.95*E* − 02	19			

**Table 6:** Predicted and experimental values of total flavonoids (TF) extraction rate under optimum conditions.

Optimum conditions			TF extraction rate (g QE^a^/100 g DW)	
Time (min)	Solid/liquid ratio (g/mL)	Temperature (°C)	Predicted	Experimental^b^
40	1 : 23.073	60	0.448	0.424 ± 0.029

^a^Quercetin equivalent. ^b^Mean ± SD (*n* = 3).

**Table 7:** Comparison of total flavonoids (TF) extraction rate with or without ultrasound treatment.

	Ultrasound treatment	Solid/liquid ratio (g/mL)	Temperature (°C)	Time	TF extraction rate (g QE^a^/100 g DW)^b^
General conditions	With	1 : 20	40	30 min	0.351 ± 0.025
	Without	1 : 20	40	30 min	0.033 ± 0.005
	Without	1 : 20	40	24 h	0.301 ± 0.011

Optimized conditions	With	1 : 23	60	40 min	0.424 ± 0.029
	Without	1 : 23	60	40 min	0.037 ± 0.006
	Without	1 : 23	60	24 h	0.229 ± 0.010

^a^Quercetin equivalent. ^b^Mean ± SD (*n* = 3).

## Data Availability

The data used to support the findings of this study are available from the corresponding author upon request.
